# The Scintigraphic Findings of Gastroesophageal Reflux in Children is Related to Body Weight?

**DOI:** 10.4021/jocmr1636w

**Published:** 2013-12-13

**Authors:** Melih Engin Erkan, Aybars Ozkan, Ayse Yilmaz, Muhammet Asik, Cemalettin Gunes, Mehmet Zeki Yilmaztekin, Ahmet Semih Dogan

**Affiliations:** a Department of Nuclear Medicine, Duzce University School of Medicine, Turkey; b Department of Pediatric Surgery, Duzce University School of Medicine, Turkey; c Department of Pediatrics, Duzce University School of Medicine, Turkey

**Keywords:** Gastroesophageal reflux disease, Scintigraphy, Body weight

## Abstract

**Background:**

Gastroesophageal reflux disease (GERD) is the most common cause of children admissions to pediatric gastroenterology unit and affects about 30% of pediatric population. Body weight and height percentiles of children with GERD and their relationship between presence and the severity of reflux on scintigraphic images were studied.

**Methods:**

Patients who underwent reflux scintigraphy between 2005 - 2012 were retrospectively reviewed. Among 200 patients, 49 patients were involved that their ages were ranging from 0 to 18 years old and body weight and height percentiles were recorded. Accurately 37 MBq (1 mCi) 99mTc-MAA in 100 - 150 mL of milk was ingested by the patient. Presence, number, duration and level of reflux were evaluated on the dynamic images. Presences of reflux within last ten minute were also recorded. Region-of-interests were drawn on esophagus and stomach and reflux ratio (RR) was calculated.

**Results:**

The ratio of the presence of reflux which occurred within the last ten minutes was significantly higher in children with low body weight percentile. High-level reflux frequency was higher in these children than in normal’s. Presence of reflux which occurred within the last ten minutes was related with low body weight percentile.

**Conclusions:**

If reflux is shown within the last ten minutes and there is high level of reflux, the clinician should be warned about possible low body weight percentile in the future and scintigraphic study should be a guide or a reference for the assessment of more effective treatment methods.

## Introduction

Gastroesophageal reflux disease (GERD) is the most frequently encountered esophageal disorder. GERD occurs with the passage of gastric contents into the esophagus, leading to a variety of symptoms and/or complications. The disease is the most common cause of neonatal admissions to pediatric gastroenterology unit and affects about 30% of pediatric population [1]. Complications can be divided into two groups as esophageal and extra esophageal. The major complications are vomiting, weight loss, dysphagia, abdominal or substernal pain, esophagitis and respiratory diseases.

Nuclear medicine methods contribute to the diagnosis and treatment of GERD in pediatric age group and provide a simple, physiologic and sensitive non-invasive procedure. It has been reported that radionuclide imaging has 15-100% sensitivity and 81-100% specificity. Both acid and alkaline reflux can be detected and allow quantitative evaluation of reflux [2-4].

Children with severe gastroesophageal reflux disease and recurrent vomiting may have delayed weight gain and growth [4]. Studies have shown that scintigraphic findings and GERD symptoms, underlying cause, medical and surgical treatment response are associated with severity and progression of disease [5-9]. However, the relationship between scintigraphic findings with low percentile of body weight is not mentioned before. In this study, we investigated body weight percentiles of children with GERD and their relationship between presence and the severity of reflux on scintigraphic images.

## Materials and Methods

Patients who had clinically gastroesophageal reflux symptoms and underwent reflux scintigraphy between 2005 and 2012 were retrospectively reviewed. Among 200 patients, we enrolled 49 patients that their ages were ranging from 0 to 18 years old and genders, reflux scintigraphy findings, body weight and height percentiles were recorded.

Radionuclide Reflux Scintigraphy; after 4 hours of fasting, accurately 37 MBq (1 mCi) 99mTc -Macroagregated albumin (MAA) in 100 - 150 mL of milk was ingested by the patient. Then 25 - 50 mL of milk was ingested to clean up residual radioactive substance remaining in the mouth and esophagus. On supine position, 64 × 64 matrix, 5-second posterior images were obtained for 30 minutes using a single detector gamma camera with a low-energy, general-purpose, parallel-hole collimator. Presence of reflux was evaluated on the dynamic images. Region-of-interests were drawn on esophagus and stomach on the frame which has most intense radiotracer uptake. Reflux ratio (RR) was calculated as RR = (esophagus activity/stomach activity) × 100 formula. Reflux levels were determined as low, medium and high. Numbers of reflux episodes within 30 minutes, duration of reflux, and presence of reflux within last ten minute were also recorded.

Statistical analyses were performed by using SPSS^®^ 12 (Statistical Package for Social Sciences software, 12, Chicago, IL, USA). Normal distribution of continuous data was tested by using the histogram curves. Mann-Whitney U test was used when comparing the continuous data in two independent groups. Continuous variables are presented as median (minimum-maximum). The chi-square test was used when comparing categorical variables in two independent groups and categorical variables were expressed as frequencies and percentages. P < 0.05 was considered as statistically significant.

## Results

The median age of the patients was 48 months (1 - 192) and male/female ratio was 26/23. Presence of reflux in girls was 46.2% (12), whereas it was 39.1% (9) in boys (P = 0.620). The median ages were 48 months (3 - 168) for children with reflux and 51 months (1 - 192) for children without reflux (P = 0.664). Body weight percentiles were similar in children with and without reflux.

Although the median age value is lower in children with low body weight percentile, this difference was not statistically significant, height Z scores of these children were significantly lower than those who had normal body weight percentiles. The ratio of the presence of reflux which occurred within the last ten minutes was significantly higher in children with low body weight percentile. High-level reflux frequency was higher in these children than in normals, with significance (Table 1).

**Table 1:** Comparison of the Scintigraphic Parameters With Low Body Weight Percentile

	< 3 percentile (N = 7)	3 - 97 percentile (N = 42)	P
Age (month)	24 (9 - 96)	60 (1 - 192)	0.424
Female gender (n, %)	4 (57.1)	22 (52.4)	0.571
Z score for height	-2.67 ((-3.60) - (-2.00))	-0.54 ((-2.80) - (4.10))	< 0.001
Reflux rate	0.58 (0 - 15.01)	0 (0 - 30.7)	0.566
Number of episodes	2 (0 - 8)	0 (0 - 6)	0.091
Presence of reflux (n, %)	4 (57.1)	17 (40.5)	0.337
Frequency of a high-level Reflux (n, %)	5 (71.4)	9 (21.4)	0.015
Presence of reflux within last ten minutes (n, %)	5 (71.4)	7 (16.7)	0.007

## Discussion

Esophageal dysfunction may cause problems which include a series of growth and developmental retardation, therefore implementation of early diagnosis and appropriate treatment is important. GERD is an esophageal dysfunction as a result of reflux of stomach contents to esophagus, leading to a series of symptoms and complications. Factors such as delayed gastric emptying, decreased lower esophageal sphincter (LES) pressure, increased frequency of transient LES relaxation, low clearance of the reflux from the esophagus, corruption of the esophageal mucosal defense often play a role in its pathogenesis [10, 11].

In diagnosis of GERD, clinic examination, upper GI endoscopy, 24-hour esophageal pH monitoring and even histological sampling methods are used. Radionuclide GER studies are used as non-invasive methods for diagnose and treatment monitoring. In addition, it allows the quantitative evaluation of the amount of gastric reflux and evaluation of the reflux level [12]. In the current study, we investigated the relationship between growth in children and scintigraphic data obtained by quantitative and visual analysis. The frequency of high level reflux and ratio of reflux which occurred in last ten minutes were significantly higher in children with low body weight percentile.

Number of children in the study group that identified growth retardation is not sufficient to achieve the desired statistical power. Prospective studies that investigate the effect of the incidence of reflux and reflux symptoms in children with growth retardation may provide more clear and reliable results.

In the literature, previous studies mentioned about relationship between reflux scintigraphy and clinical symptoms and role of the reflux in etiology of respiratory disease [3, 5]. According to our knowledge, although it is well known that growth and development are affected by clinically [3, 12-15]. Severe reflux disease that accompanied with vomiting, findings of reflux scintigraphy has not yet been compared with objective parameters of growth such as body weight percentile. In addition, reflux that occurs when gastric content volume is low is clinically more important because in these patients reflux occurs without increased intragastric pressure and acid buffering [16]. In this respect, reflux within the last 10 minutes is crucial, and in our study, it has related with low body weight percentile. In conclusion, if reflux is shown within the last ten minutes and there is high level of reflux, the clinician should be warned about possible low body weight percentile in the future and scintigraphic study should be a guide and a reference for the examination of more effective treatment methods.
